# Using 3–6 differences in essential fatty acids rather than 3/6 ratios gives useful food balance scores

**DOI:** 10.1186/1743-7075-9-46

**Published:** 2012-05-24

**Authors:** Bill Lands, Etienne Lamoreaux

**Affiliations:** 1 Retired, Fellow of ASN, 6100 Westchester Park Drive, Apt.1219, College Park, MD, 20740, USA; 2 Computer Specialist, NIAAA, NIH, 5635 Fishers Lane, Bethesda, MD, 20892-8304, USA

**Keywords:** Arachidonic cascade, Calories, Cardiovascular, Essential fatty acids, Health risk assessment, Highly unsaturated, Inflammatory, Omega-3, Omega-6, Polyunsaturated

## Abstract

**Background:**

The vitamin-like omega-3 and omega-6 essential fatty acids are converted in the body to a large family of hormones which act at selective receptors that occur on nearly every cell and tissue. A relative omega-3 deficit allows overabundant actions of omega-6 hormones to develop into health disorders. People need simple, explicit information on the balance of essential fatty acids in their foods to avoid accumulating unintended imbalances in their tissue omega-3 and omega-6 fatty acids.

**Results:**

We developed an Omega 3–6 Balance Food Score that summarizes in a single value the balance among eleven omega-3 and omega-6 essential fatty acids in a food. The value allows a quantitative estimate of the impact of each food item on the proportions of omega-3 and omega-6 that will accumulate in the 20- and 22-carbon highly unsaturated fatty acids of blood, which is an important health risk assessment biomarker.

**Conclusions:**

The impact of an individual food item upon a useful health risk assessment biomarker is easily evident in a simple, explicit value for the balance among eleven essential fatty acids nutrients. Foods with more positive Omega 3–6 Balance Food Scores will increase the percent of omega-3 in the biomarker, whereas those with more negative Scores will increase the percent of omega-6 in the biomarker.

## Background

Eighteen years after the initial discovery that omega-3 and omega-6 essential fatty acids form a large family of hormones, the 1982 Nobel Prize in Physiology or Medicine recognized the importance of those hormones. The omega-3 and omega-6 forms compete with each other during the metabolic steps by which they accumulate in our tissues. Once there, they act differently in selectively forming hormones that act selectively on receptors which are present on nearly every cell and tissue in the body [1]. The pharmaceutical industry has invested billions of dollars to develop and market treatment agents that suppress excessive formation and action of the hormones formed from the omega-6 arachidonic acid by the “arachidonate cascade”. Those omega-6 hormones mediate many signs and symptoms of diverse chronic diseases and disorders. In contrast, essential omega-3 fatty acids may have beneficial actions in part by a preventive displacing competition with the omega-6 compounds [2-4].

Biomedical knowledge provides two major ways to decrease health-related problems from overabundant actions of the “arachidonate cascade”: (a) informed nutrition choices that prevent imbalances from developing into disease and (b) pharmaceutical treatments that lower the disease signs and symptoms caused by such nutrient imbalances. To help consumers make better nutritional choices, food marketers provide “Nutrient Facts” labels to inform the public of the kilocalories of metabolic energy in carbohydrate, protein and fat as well as the essential nutrients and vitamins in a defined “serving” of the food item. In addition, people need explicit information to avoid accumulating unintended imbalances in their tissue omega-3 and omega-6 fatty acids. This report describes a simple new measure of nutrient balance that predicts a food’s ability to prevent omega-3 imbalances in our tissues and allows informed personal food choices.

### Early diet-tissue quantitation

Dietary 18-carbon polyunsaturated fatty acids (PUFA) maintain the proportions of 20- and 22-carbon highly unsaturated fatty acid (HUFA) hormone precursors that are accumulated in tissues. Knowing this metabolic interaction gives insight for a preventive nutrition strategy based on the health risk assessment biomarker, the%n-6 in tissue HUFA (Equation 1). The interactions of dietary omega-3 (n-3) and omega-6 (n-3) PUFA first reported by Mohrhauer and Holman [5,6]

Equation 1. Describing the% n-6 in tissue HUFA.

(1) $$\%\text{ n}-6\text{ in HUFA}=\frac{100\text{ x }\left[\text{ n}-6\text{ HUFA }\right]}{\left[\text{n}-\text{3HUFA }+\text{n}-\text{6HUFA }+\text{n}-\text{9HUFA }\right]}$$

were confirmed with a quantitative empirical relationship that fit competitive hyperbolic interactions which maintain the% n-6 in tissue HUFA of laboratory rats [7]. The simple hyperbolic relationship for the interactions of dietary PUFA was then extended to describe the combined impact of the daily percent of food energy (en%) in dietary PUFA and HUFA on the accumulated proportions of omega-3 and omega-6 in tissue HUFA of rats, mice and humans [8]; see Equation 4 in Methods.

When more quantitative dietary data with humans became available, three of the eight constants were revised slightly to give a better fit with all of the combined results [4,9]. The empirical equation and constants reliably use daily nutrient intakes (as en%) to estimate quantitatively the likely% n-6 in HUFA maintained in plasma, red cells and whole blood [10-12]. A literature search and analysis [13] showed that Equation 4 estimates with a correlation coefficient of 0.73 (P = 0.0000) the observed tissue HUFA proportions maintained by daily en% intakes for 92 subject groups in 34 different published studies.

During the past decade, Equation 4 was put into a small spreadsheet [14] for planning and evaluating new dietary interventions. It was also put into an interactive personalized menu planning software, KIM-2 [15], to help individuals make informed choices using the nutrient data for thousands of food items that are listed in the USDA Nutrient Database [16]. The software manages eleven 18-, 20- and 22-carbon omega-3 and omega-6 acids in four categories: omega-6 PUFA (“short 6”; 18:2 and 18:3), omega-3 PUFA (“short 3”; 18:3 and 18:4), omega-6 HUFA (“long 6”; 20:3, 20:4, 22:4 and 22:5) and omega-3 HUFA (“long 3”: 20:5, 22:5 and 22:6). As noted in the Methods section, the software sums the milligrams of these four categories of essential fatty acid in all food items for a selected daily menu plan and expresses the daily intake of the categories as a percent of overall daily food energy (en%). It then combines daily en% values with Equation 4 to estimate a likely value for the health risk assessment marker, the% n-6 in blood HUFA.

Although the estimates of daily food impacts are successful [13], people find it tiresome to calculate all of the en% values for a full day’s menu when they only want to learn the impact of an individual food. As a result, we sought a new way to estimate a food’s impact by converting the balance among the milligrams per calorie of eleven dietary 18-, 20-. and 22-carbon omega-3 and omega-6 acids into a single value for each food item. This new approach to balance uses arithmetic differences of (n-3) - (n-6) rather than ratios of (n-3)/(n-6).

## Results

### Differences between short- and long-chain acids

Recognizing that many researchers have found that dietary HUFA affect tissue HUFA proportions more than dietary PUFA do, we first sought an empirical scaling factor that would allow Equation 2 to give daily menu balance values over a range from approximately −10 to +10.

(2) $$\begin{array}{l} \text{Daily menu balance}=\left(\text{en}\%\text{ short}3-\text{en}\%\text{ short}6\right)\\ \;+(\text{factor})\\ \;\times \left(\text{en}\%\text{ long }3-\text{en}\%\text{ long}6\right) \end{array}$$

**Figure 1:**
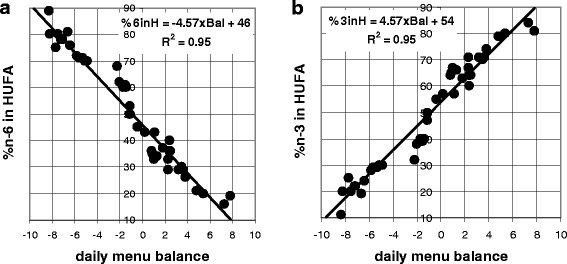
**Relationship of daily menu balance to the software-estimated HUFA proportions. (a)** %omega-3 in tissue HUFA = 4.57 × daily menu balance + 54; **(b)** %omega-6 in tissue HUFA = − 4.57 × daily menu balance + 46.

**Figure 2:**
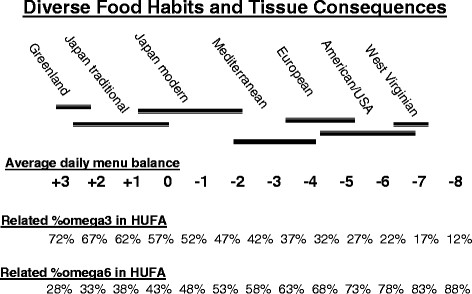
Diversity in daily food habits causes diverse consequences for blood HUFA proportions.

Using daily en% values from 48 very different daily menu plans like those stored in the KIM-2 software [15], we found that a value of 7 fit that goal. With this factor, we saw daily menu balance values calculated from en% values in the diverse menu plans correlated well with the values for the blood biomarker,% n-6 in HUFA, estimated by the KIM-2 software (Figure 1). The 4.6 value of the slope of the correlation indicates that each integer more positive in the average daily menu balance value gives about a 5% higher proportion of omega-3 in blood HUFA. In this context, the proportions of 30 to 40% n-6 in HUFA associated with eating traditional Japanese foods (that have an average daily menu balance near +1) are lower than the 60% n-6 in HUFA associated with an average Mediterranean diet that has an average daily balance near −3 (Figure 2). The very wide range of ethnic food habits worldwide maintains average daily menu balance values that range from +3 to −8. That diversity in typical daily foods causes the wide range of HUFA proportions (28% to 88% n-6 in HUFA) that has been reported for different populations [3,13,17-19]. 

### Shifting from daily balance to a food item’s balance

To generate an Omega 3–6 Balance Score for each individual food item, we replaced the dimension of the daily en% [100 × (total mg) × (.009Cal/mg)/(total Cal)] used in Equation 2 with the closely-related dimension of mg/Cal for each food item as shown in Equation 3.

(3) $$\begin{array}{l} \text{Omega }3-6\text{ Balance Score}\\ \;=\left(\text{mg short}3-\text{mg short}6\right)/\text{Cal}+7\\ \;\times \left(\text{mg long }3-\text{ mg long}6\right)/\text{Cal} \end{array}$$

**Figure 3:**
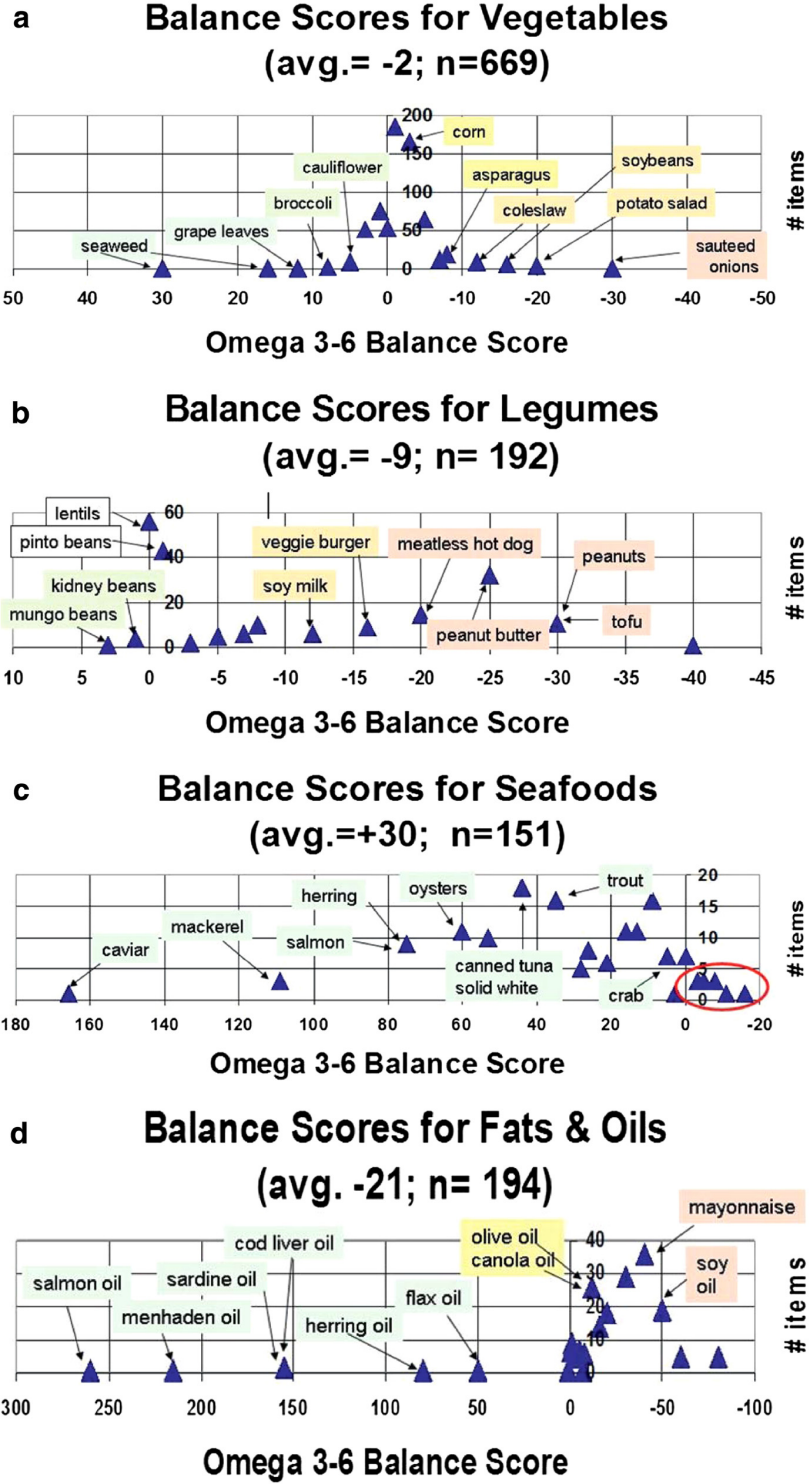
**Diversity in Omega 3–6 Balance Food Scores.** The ordinate axis notes the frequencies of occurrence for food items with various Omega 3–6. Balance Food Score values indicated on the abscissa for vegetables **(a)**, legumes **(b)**, fish and seafood **(c)**, fats and oils **(d)**.

The resulting Score characterizes the balance of essential fatty acids in each food item independent of any other foods that might be eaten during the day. Table 1 shows that the average Scores for the twenty four different food groups used by the USDA Nutrient Database SR24 [16] ranged from −21 to +30. While the average Score for fruits and vegetables is near zero, it is very negative for the fats and oils group and very positive for the fish and seafood group. In fact, negative Scores in the latter group are almost all due to the impact of food oils that have been added to the fish or seafood items. The negative values for canned tuna in vegetable oil (−9), tuna salad (−16) and fried breaded shrimp (−11) are circled in Figure 3c. The overall average Omega 3–6 Balance Score for all 5,100 food items is about −5. 

**Table 1:** Average Omega 3–6 Balance Food Scores (overall avg. = −5; n = 5,108)

**USDA Food Group**	**Omega 3–6 Balance**
**(n = items in group)**	**(average value)**
Cereals, Grains & Pasta (n = 159)	- 3
Breakfast Cereals (n = 291)	- 3
Baked Products (n = 410)	- 6
Vegetables (n = 669)	- 2
Fruits & Fruit Juices (n = 286)	- 1
Dairy & Egg Products (n = 189)	- 2
Poultry Products (n = 204)	- 11
Pork Products (n = 193)	- 7
Beef Products (n = 388)	- 3
Lamb, Veal, & Game (n = 196)	- 5
Sausages & Lunch Meats (n = 210)	- 7
Legumes (n = 192)	- 9
Fish & Seafoods (n = 151)	+30
Nuts & Seeds (n = 133)	- 18
Fats & Oils (n = 194)	- 21
Soups, Sauces, & Gravies (n = 200)	- 5
Snacks (n = 126)	- 12
Fast Foods (n = 261)	- 8
Meals & Entrees (n = 28)	- 5
Restaurant Foods (n = 52)	−12
Sweets (n = 147)	- 3
Beverages (n = 99)	- 1
Spices & Herbs (n = 48)	- 2
Baby Foods (n = 324)	- 6

A closer look at the frequency of 669 Food Scores for vegetables (Figure 3a) shows hundreds of vegetable items have Scores near zero (e.g., cabbage, potatoes, onions). Again, the negative scores for cole slaw (−13), potato salad (−21) and sautéed onions (−31) reflect the impact of food oils that have been added to the vegetable items. Calorie-weighted average scores are analogous to en% values in daily menu plans in the existing interactive KIM-2 software [15]. When many different food items are combined on a calorie-weighted basis to form an average daily menu balance from the diverse positive and negative Scores, the result is an average daily value between −10 and +10. Calorie-weighted average scores are related to en% values in daily menu plans of the existing interactive KIM-2 software [15]. Typical current American food choices have average values around −6 to −7. Inspection of the Scores in Figure 3 readily identifies foods that can combine to make a daily average more negative or more positive.

## Discussion

The wide impact on health of a relative omega-3 deficit reflects the multiple actions of selective hormone receptors which respond differently to n-3 and n-6 hormones [1]. As a result, unintended dietary imbalances that cause imbalance among tissue precursors of hormone actions have selective effects on nearly every cell and tissue in the human body and influence many aspects of human physiology and pathology [18]. The list of health problems related to omega-3 deficits with elevated proportions of omega-6 in the hormone precursors has grown to include atherosclerosis, thrombosis [19], arrhythmia, heart attacks, stroke, immune-inflammatory disorders, asthma, arthritis, cancer proliferation [18], obesity [20], psychiatric disorders, depression, suicide, homicide [21,22], oppositional behavior, unproductive workplace behaviors, length of stay in hospitals [23] and annual healthcare claim costs [24].

Health risk assessment (HRA) with a simple low-cost finger-tip blood sample [10-12] informs individuals of their personal essential fatty acid status. Such test results relate quantitatively to the risk for cardiovascular mortality (death = 3 × (%n-6 in HUFA) - 75; [17]). The biomarker value of the% n-6 in HUFA links the balance of n-3 and n-6 acids in daily foods to the risk of many aspects of immune-inflammatory and cardiovascular disease [17,18].

The associated health claim costs for disorders linked to omega-3 deficits [18-24] are predictably less as people voluntarily choose foods that lower their HRA value from its current USA average level near 80% n-6 in HUFA to a lower value near 60%. Figure 2 illustrates that such a lowering represents changing the average daily Omega 3–6 Balance Food Score from −7 to −3. Figure 3A illustrates that a majority of vegetables have Scores more positive than the typical daily American average balance near −6 or −7.

The Omega 3–6 Balance Scores rapidly and easily identify food items that can move a person’s daily average Food Score from −7 to −3 or to an even more positive value. For example, combining a tablespoon of flaxmeal (+32) with a half cup of oatmeal (−4) or adding flax oil (+46) to canola oil (−11) helps maintain a more positive overall food balance. Similarly, peanut butter (−24) with added flax meal and oil has a more positive score. Consumers can readily see that eating farmed (+28) or wild (+73) grilled salmon can do much to providing a more positive overall daily food balance.

The ease with which Omega 3–6 Scores help interpret the impact of foods is illustrated with the top 100 foods from a USDA Key Foods list [25] based on NHANES 2007–08 intake data [26]. The un-weighted average Score of the 100 items is about −6, equivalent to an HRA value of 78% n-6 in HUFA (commonly reported for Americans). None of the 100 items was a seafood. When the ten most negative food items are removed, the un-weighted average Score of the remaining 90 items is about −3, equivalent to an HRA value of 60% n-6 in HUFA (commonly associated with a Mediterranean diet). Traditional Mediterranean foods do not include the ten items removed: soybean oil, -50; mayonnaise, -46; tub margarine, -39; microwave popcorn, -37; “Italian” salad dressing, -35; potato chips, -29; stick margarine, -28; vegetable shortening, -28; peanut butter, -24; tortilla chip snacks, -24. However, they do include some seafood items that would move the daily overall average to values more positive than −3.

The rise in dietary omega-6 intake caused by added food oils in USA food supplies during the 20^th^ century [27] has been associated with a rise in the prevalence of many chronic disorders. As a result, much discussion now addresses dietary approaches that can lower the preventable risk for these serious health problems. Recent comments [28] have emphasized the need to account fully for all n-3 and n-6 dietary intakes to avoid mis-interpreting the outcomes from large clinical trials.

Until now, concerns over unwanted balances among n-3 and n-6 essential fatty acids have often addressed the concept of n-3/n-6 ratios without providing an explicit estimate of how such ratios of fatty acids in foods quantitatively impact the balance of HUFA accumulated in tissues. We believe that the new explicit Omega 3–6 Balance Food Scores can help people easily identify and make informed food choices that lower their personal health risk assessment biomarker value.

### Limitations of diet and risk assessment

Attempts to describe quantitative abundances of nutrients in foods eaten are confounded by varietal and seasonal differences in nutrient composition, imprecise recall of quantities eaten and highly diverse intakes in meals from day-to-day and week-to-week. Nevertheless, estimates are useful for predicting how the amounts of vitamin-like n-3 and n-6 nutrients in our food [16] affect average proportions of n-3 and n-6 hormone precursors accumulated in our body [13]. Quantitative descriptions of accumulated n-3 and n-6 hormone precursors are confounded by diverse modes of recording and reporting tissue composition [12]. Finally, relating relative tissue abundances to receptor-mediated health outcomes is confounded by diverse biomarkers used to characterize health risk. Biomarkers that are only predictive of harm and are not factors mediating harm have distracted attention and resources away from decreasing preventable mediators during primary prevention [24,29]. The earlier developed interactive planning tool, KIM-2, successfully links key variables for a single day’s intake. However, some people want only to consider the impact of a single food in overall health conditions. Omega 3–6 Balance Scores were developed as a tool for them to evaluate a single food independent of any other food that may be eaten. Calorie-weighted average scores for a day’s combined food are analogous to en% values in daily menu plans of the existing interactive KIM-2 software. Figure 1 shows how a day’s calorie-weighted average score predicts the likely%n-6 in HUFA using 46–4.57 × calorie-weighted average. Thus, the 3–6 differences among essential fatty acids (rather than 3/6 ratios) give a useful tool to use in discussing a food’s contribution to health.

## Conclusions

A simple, explicit description of the different abundances of all n-3 and n-6 nutrients in a food makes their impact on an important health risk assessment biomarker easily evident. Eating foods with more positive Omega 3–6 Balance Food Scores increases the estimated percent of omega-3 in tissue HUFA, whereas foods with more negative Scores increase the omega-6 percent.

## Methods

### Differences for short- and long-chain acids

We used the interactive menu planning software, KIM-2 (which contains data from the USDA Nutrient Database SR15) to design 48 very different individual daily menu plans that fit different lifestyles and daily energy requirements. The food choices in each plan were estimated to give health risk assessment biomarker values that ranged from 15%n-6 in HUFA to 89%n-6 in HUFA. The KIM-2 software groups eleven 18-, 20- and 22-carbon omega-3 and omega-6 acids into four categories: omega-6 PUFA (“short 6”; “P_6_”; 18:2 and 18:3), omega-3 PUFA (“short 3”; “P_3_”; 18:3 and 18:4), omega-6 HUFA (“long 6”; “H_6_”; 20:3, 20:4, 22:4 and 22:5) and omega-3 HUFA (“long 3”: “H_3_”; 20:5, 22:5 and 22:6). It sums the milligrams of these four categories of fatty acid for all food items in each daily menu plan and expresses the sum as a percent of overall daily food energy (en%). It then uses the daily en% values with Equation 4 [9] to estimate a likely value for the health risk assessment biomarker,% n-6 in blood HUFA. The constants currently used with Equation 4 are: HC_3_ = 3.0, HC_6_ = 0.70, PC_3_ = 0.0555, PC_6_ = 0.0441, HI_3_ = 0.005, C_O_ = 5.0, Ks = 0.175.

Equation 4 Estimating the%n-6 in HUFA predicted from en% of dietary n-3 and n-6 acids.

(4) $$\begin{array}{l} \text{Predicted }\%\text{ n}-6\;\text{in HUFA}=\frac{100}{1+\left({\text{HC}}_{6}/\text{en}\%{\text{H}}_{6}\right)\left(1+\text{ en}\%{\text{H}}_{3}/{\text{HC}}_{3}\right)}\\ \;+\frac{100}{1+\left({\text{PC}}_{6}/\text{en}\%{\text{P}}_{6}\right)\left(1+\text{en}\%{\text{P}}_{3}/{\text{PC}}_{3}+\text{en}\%{\text{ H}}_{3}/{\text{HI}}_{3}+\text{en}\%\text{O}/C_{o}+\text{en}\%P_{6}/\text{Ks}\right)} \end{array}$$

The daily en% values for the four categories in the 48 different menu plans were alternatively combined in Equation 2 to allow trial-and-error tests that empirically determined that a factor of 7 gives average daily menu scores that range approximately from −10 to +10. Figure 1 shows the relationship between the average daily menu score when en% values were used with 7 in Equation 2 compared to the resultant health risk assessment biomarker,% n-6 in blood HUFA, when en% values were used in Equation 4 by the KIM-2 software.

### Calculating a food item’s omega 3–6 balance

Data from the USDA Nutrient Database SR24 [16] were entered into FileMaker Pro 11, and 5,100 food items were selected from the initial 13,200 items by deleting redundant servings and examples of brain tissue or raw meats not likely to be widely eaten. Omega 3–6 Balance Scores were calculated using Equation 3. Some of the more recent data in the USDA Nutrient Database identify specific fatty acids rather than designating a peak as “undifferentiated”. Specific acid values were used when available, otherwise the “undifferentiated” value was used. The resulting 5,100 Omega 3–6 Balance Food Scores were grouped into the twenty four USDA-defined food groups that had average Scores ranging from −21 to +30***,*** as shown in Table 1. Data sets of all the Food Scores supporting the results of this article are available as searchable pdf files in a repository of Omega 3–6 Balance Food Scores posted at http://www.fastlearner.org/Omega3-6Balance.htm. The Omega 3–6 Balance Scores can be downloaded as a free “app” for mobile devices from http://www.fastlearner.org/Omega3-6BalanceApp.htm to help guide personal food choices when shopping or preparing meals.

## Abbreviations

HRA: Health risk assessment; HUFA: Highly unsaturated fatty acid; n-3: Omega-3; n-6: Omega-6; PUFA: Polyunsaturated fatty acid.

## Competing interests

The authors declare that they have no competing interests.

## Authors’ contributions

BL developed the text and equations, and EL developed the software for KIM-2 and the calculations described in this manuscript. Both authors read and approved the final manuscript.
